# Ginsenoside compound K reduces neuronal damage and improves neuronal synaptic dysfunction by targeting Aβ

**DOI:** 10.3389/fphar.2023.1103012

**Published:** 2023-02-16

**Authors:** Na Li, Qihang Pang, Yanhong Zhang, Jianan Lin, Hui Li, Zhen Li, Yaxin Liu, Xingyu Fang, Yu An, Haonan Bai, Dianyu Li, Zhanhong Cao, Jian Liu, Qing Yang, Shaodan Hu

**Affiliations:** ^1^ Changchun University of Chinese Medicine, Changchun, China; ^2^ Department of General Surgery, Qian Wei Hospital of Jilin Province, Changchun, China

**Keywords:** ginsenoside compound k, amyloid β, neuronal damage, synaptic function, Alzheimer’s disease

## Abstract

**Background:** Alzheimer’s disease (AD) is the most common neurodegenerative condition worldwide, with amyloid *ß* (Aβ) fibrils presenting as its main pathological feature. This study investigated whether Ginsenoside Compound K (CK) has activity against Aβ and its mechanism in reducing synaptic damage and cognitive impairment.

**Methods:** The binding capacity of CK to Aβ42 and Nrf2/Keap1 was determined using molecular docking. Transmission electron microscopy was used to monitor CK-mediated degradation of Aβ fibrils. The effect of CK on the survival of Aβ42-damaged HT22 cells was determined using a CCK-8 assay. The therapeutic efficacy of CK in a scopoletin hydrobromide (SCOP) induced cognitive dysfunction mouse model was measured using a step-down passive avoidance test. GO enrichment analysis of mouse brain tissue was peformed using Genechip. Hydroxyl radical scavenging and reactive oxygen species assays were performed to verify the antioxidant activity of CK. The effects of CK on the expression of Aβ42, the Nrf2/Keap1 signaling pathway, and other proteins were determined by western blotting, immunofluorescence, and immunohistochemistry.

**Results:** Molecular docking results showed that CK interacts with Lys16 and Glu3 of Aβ42. CK reduced the aggregation of Aβ42 as observed using transmission electron microscopy. CK increased the level of insulin-degrading enzyme and decreased the levels *ß*-secretase and γ-secretase; therefore, it can potentially inhibit the accumulation of Aβ in neuronal extracellular space *in vivo*. CK improved cognitive impairment and increased postsynaptic density protein 95 and synaptophysin expression levels in mice with SCOP-induced cognitive dysfunction. Further, CK inhibited the expression of cytochrome C, Caspase-3, and cleaved Caspase-3. Based on Genechip data, CK was found to regulate molecular functions such as oxygen binding, peroxidase activity, hemoglobin binding, and oxidoreductase activity, thus affecting the production of oxidative free radicals in neurons. Further, CK regulated the expression of the Nrf2/Keap1 signaling pathway through its interaction with the Nrf2/Keap1 complex.

**Conclusion:** Our findings show that CK regulates the balance between Aβ monomers production and clearance, CK binds to Aβ monomer to inhibits the accumulation of Aβ, increases the level of Nrf2 in neuronal nuclei, reduces oxidative damage of neurons, improves synaptic function, thus ultimately protecting neurons.

## 1 Introduction

Alzheimer’s disease (AD) is a neurodegenerative disease and is the most common form of dementia (West and Bhugra, 2015). Cognitive dysfunction is a pre-AD manifestation, followed by progressive deterioration in behavior and mood (Garcia-Ptacek et al., 2016). The main pathological manifestations of AD are senile plaques and neurofibrillary tangles (Inestrosa et al., 2011). The senile plaque is mainly formed by the massive accumulation of *ß*-amyloid (Aβ) in the hippocampus. Aβ was formed by *ß*-amyloid precursor protein (APP) shearing by secretase, secretase mainly includes α-secretase (Lichtenthaler, 2012), *ß*-secretase (BACE1) (Spoelgen et al., 2006) and γ-secretase (PS1) (Takahashi et al., 2008). APP is cleaved by BACE1 and PS1 to form neurotoxic Aβ (Tyan et al., 2012), formation of Aβ fibres and oligomers in extraneuronal and neuronal space, produce neurotoxicity and leading to neuronal synaptic damage (Sadigh-Eteghad et al., 2015).

Ginseng is a traditional Chinese medicine that contains multiple active ingredients, including Ginsenoside (Mancuso and Santangelo, 2017). Ginsenosides are divided into two subtypes, *viz*. Protopanaxadiol (PPD) and protopanaxatriol (PPT) (Smith et al., 2014). PPD compounds possess various biological properties and have neuroprotective effects (Lu et al., 2018), but not easily absorbed by the body (Kim and Kim, 2018). Ginsenoside Compound K (CK) was the main metabolite of ginsenosides *in vivo* (Lee et al., 2013), and is obtained following the metabolism of ginsenoside by the intestinal flora (Yang et al., 2015). CK has good pharmacological activity; it was reported to inhibit neuronal damage associated with Aβ and improve learning memory in mice through its antioxidative properties (Oh and Kim, 2016).

Nevertheless, to date, the mechanism behind how CK regulates the aggregation of Aβ and protects neurons is still unclear and needs elucidation. Therefore, in this study, we first investigated the ability of CK to regulate Aβ aggregation *in vitro* and *in vivo*. Then screened the protective pathway of CK on neurons by sequencing the brain tissue from SCOP-induced memory-impaired mice, and further validation the protective effect of CK using memory-impaired mice and Aβ-damaged HT22 cells.

## 2 Materials and methods

### 2.1 Transmission electron microscopy

CK (Chengdu Desite Biotech Co., Ltd., China, Purity: ≥90%) solution was dissolved in Aβ42 (Jill Biochemical (Shanghai) Co., Ltd., China, Purity: 95.20%) monomer solution to obtain final concentrations of 0 μM, 50 μM, 100 μM, and 200 μM, the final concentration of Aβ42 was 50 μM. Aβ42 monomer solution was incubated alone or with CK for 5 days at 37°C. The Aβ42 monomer and treated samples were dropwise to glow-discharged carbon-coated 300-mesh copper grids, were adsorbed for 10 min and blotted with filter paper, and air-dried. Images were examined with a JEM-1230 electron microscope operated at 80 keV.

### 2.2 Hydroxyl radical scavenging

Two hundred microliters (200 μL) of different concentrations of CK (1 mg/mL, 2 mg/mL, 4 mg/mL, 8 mg/mL) were mixed with 200 μL H_2_O_2_ (Xilong Chemical Co., Ltd., China) (6 mM) and 100 μL FeSO_4_ (Liaoning Quan Rui Reagent Co., Ltd., China) (6 mM), The mixture was stirred and mixed for 10 min. Next, the resultant mixture was added 200 μL salicylic acid (Beijing Yongding Chemical Factory, China) (6 mM) maintained at 37°C for 25 min. The absorbance of the resultant mixture was measured at 510 nm and the following equation was used to determine radical scavenging rate:

$$Scavenging\;rate=\left[1-\left(A_{1}-A_{2}\right)\;/\;A_{0}\right]\times 100\%.$$

A_1_ is the absorbance of CK mixed with the reaction solution. A_2_ is the absorbance measured with distilled water instead of H_2_O_2_. A_0_ is the absorbance of CK replaced by distilled water.

### 2.3 Molecular docking

The 3D structure SDF file of ginsenoside CK was first obtained from the PubChem database. File format conversion to PDB was peformed using Discovery Studio 2019 software. The protein structures of Aβ, Nrf2-Keap1, and the receptor protein were downloaded from the RCSB PBD database (https://www1.rcsb.org/). The https://swift.cmbi.umcn.nl/servers/html/prepdock.html website was used to manipulate receptor proteins, including correcting side chains and water molecules. The receptor protein and ligand drug molecules were de-watered and non-polar hydrogen atoms were added using AutoDock Tools 1.5.6 software to construct a docking site cavity. After docking was complete, the best docking conformation was selected for verification based on the principles of conformational rationality and low energy, and the docking results were visualised using PyMOL 2.2.0 software.

### 2.4 Mice and drug treatents

ICR mice (weighing 25–30 g) were purchased from Changchun Yisi Experimental Animal Co., Ltd. (China). SCOP was purchased from Shanghai Yuanye Biotechnology Co., Ltd. (China). Memantine Hydrochloride (MH) was purchased from Adamas Reagent Co., Ltd. (China). Mice were housed in a sterile environment with controlled room temperature at 22°C–25°C and allowed food and water. All experiments were approbed by the Changchun University of Chinese Medicine Animal Ethics Committee. Mice were randomly divided into three groups, with 10 mice in each group: Control group (0.9% Saline), SCOP group (SCOP 2 mg/kg), MH group (Memantine Hydrochloride, MH 3 mg/kg + SCOP 2 mg/kg), CK1 group (CK 20 mg/kg + SCOP 2 mg/kg), CK2 group (CK 40 mg/kg + SCOP 2 mg/kg). The Control group, MH group, CK group were administered saline (i.g), MH (i.g), CK (i.g) once daily for 14 consecutive days. Starting from the 7th day, except in the case of control group mice, SCOP was injected intraperitoneally, 30 min after treatment with the corresponding drug.

### 2.5 Novel object recognition

A three day NOR paradigm was used with an open field measuring 40 cm (w) × 40 cm (L) × 40 cm (h). On day 1, all mice were habituated to the empty opaque plastic chamber for 10 min. After 24 h of familiarization, mice were placed in the chamber with two identical objects and allowed to explore for 10 min. A testing trial occurred on day 5 and one object was replaced with a randomly chosen novel object, and the mice allowed to explore for 10 min. Object exploration was defined when the nose of the mice directed towards the object at a distance less than 2 cm and measured by the discrimination index which indicates the difference of time spent between a novel (TN) and familiar object (TF). It was calculated using the total amount of time spent with both objects in the test phase [Discrimination Index = TF/(TN + TF)].

### 2.6 Step-down passive avoidance test

The Step-Down Passive-Avoidance (SDPA) test was detected the learning and memory ability of mice by passively avoiding electrical stimulation. An insulated platform was placed in the reaction chamber where the bottom grid floor can be energized. During the training, the mice was subjected to an electric shock on the grid floor, and mice were eventually trained to stay on the platform. Retention tests were carried out 24 h after the traning session. Placed the mice on the platform and recorded the latency and number of errors within 5 min. After behavioral testing, the mice were anesthetized and sacrificed. The brain tissue was quickly removed and placed on ice, washed with PBS buffer solution, and some brain tissues were stored at −80°C for subsequent RNA extraction and microarray analysis, and some brain tissues were placed in 4% paraformaldehyde for subsequent immunohistochemistry.

### 2.7 Immunohistochemical

Mouse brain tissue is dehydrated and embedded in paraffin, paraffin sections of brain tissue were dewaxed and hydrated, and incubated in 3% H_2_O_2_ to eliminate endogenous peroxidase activity. Slides were washed in PBS and sections were incubated overnight for rabbit anti-beta Amyloid Antibody (BOSTER, China), rabbit anti-IDE (Proteintech, China), rabbit anti-BACE1 (Proteintech, China), APP (Cell Signaling Technology, United States ), rabbit anti-PS1 (Bioss, China) at 4°C. Sections were incubated with biotinylated goat anti-rabbit lg-G for 30 min at room temperature. Slices were incubated in DAB (ZSGB-Bio, China) stain for 5–8 min until a brown precipitate is produced. Image observation and analysis using ImageScope software.

### 2.8 RNA isolation and microarray analyses

Control group, SCOP group, and CK2 group of mice were sacrificed, and brains were isolated as described earlier. And total RNA was purified using Trizol as per manufacturer’s protocol. Evaluate RNA integrity was using the Agilent 2,100 Bioanalyzer with an RNA LabChip Kit (Agilent Technologies). cDNA was prepared from total RNA using a random priming method followed by fragmentation of double-stranded cDNA, labelling and hybridization onto the GeneChip WT Terminal Labeling and Controls Kit. Microarrays were scanned with the Affymetrix GeneChip Scanner 3,000 7G. Raw data using Affymetrix GeneChip Operating Software. The obtained data were screened, and the differentially expressed genes were analyzed by unsupervised hierarchical clustering and displayed as heatmap. The GO Enrichment Analysis method uses the differential gene to perform gene function annotation based on the GO database (http://www.geneontology.org). Obtain the molecular function of the gene, and then screen out the significant function of the gene. Calculate the significance level (*p*-value) and false positive rate (FDR) of each function using Fisher’s exact test and multiple comparison test. The standard of significant screening: *p*-value < 0.01.

### 2.9 Preparation of amyloid

Lyophilized Aβ42 was reconstituted in 100% 1,1,1,3,3,3 hexafluoro-2-propanol (HFIP) (Shanghai Macklin Biochemical Technology Co., Ltd., China) to make a 1 mM dispersion. The mixture was incubated at 25°C for 60 min, placed on ice for between 5 and 10 min, moved to a fume hood to evaporate the HFIP, and subsequently formation of an Aβ peptide film occurred after air-drying. The film was resuspended in 5 mM anhydrous DMSO, and PBS was added to dilute the mixture to a 1 mM concentration.

### 2.10 Cell culture and treatment

Mouse hippocampal HT22 cells were obtained from Shanghai Enzyme Research Biotechnology Co., Ltd. (Shanghai, China). Cultured in high glucose DMEM supplemented with 10% FBS at 37°C in a water-saturated atmosphere of 5% CO2 incubator (Thermo, United States). Cells were subcultured 3 times a week. All experiments were performed after the cells were attached for 24 h, Cells treated with different concentrations of CK (2.5, 5, 10 μM) for 24 h, In addition to Control, cells treated with 10 μM Aβ42 for 24 h.

### 2.11 Measurement of cell viability

Detection of cell viability using CCK-8 (Dojindo Molecular Technologies, INC., Japan) assay. HT22 cells were cultured in 96-well plates at 1 × 10^5^ cells/mL concentration. Treated cells were added 10 μL CCK-8 per well. Determination of absorbance at 450 nm using microplate reader (TECAN, Switzerland) and calculated cell viability.

### 2.12 Measurement of intracellular ROS

Treated cells were added DCFH-DA diluted in serum-free medium at a ratio of 1:1,000, Incubate at 37°C for 20 min, Observing the fluorescence expression of ROS under fluorescence microscope (Leica, Germany). Fluorescence intensity was measured by fluorescence microplate reader (TECAN, Switzerland) at 485 nm excitation and 525 nm emission.

### 2.13 Immunofluorescence assay

Treated cells were rinsed in PBS and permeabilized with 4% paraformaldehyde for 15 min. The cells were added 0.5% Triton X-100 at room temperature for 20 min after rinsing with PBS. The cells were incubated with primary antibody anti-rabbit Nrf2 (Proteintech, China) overnight at 4°C. After incubation, HT22 cells were rinsed three times with PBS and incubated with specific secondary antibody for 1 h at room temperature. The cells were incubated with DAPI for 5 min at room temperature to stain nucleus. Photographing and observing with a laser confocal microscope (Olympus, Japan).

### 2.14 Western blot analysis

Cell lysed protein were eletrophoretically separated on 15% SDS-PAGE and transferred to PVDF membranes (Millipore, United States). The membranes were incubated with 5% skim milk for 1 h to block non-specific binding protein, and reacted with primary antibody at 4°C overnight. Afterwards, the membranes were incubated with secondary antibody conjugated with goat anti-rabbit (Proteintech, China) or goat anti-mouse HPR (Proteintech, China) at room temperature for 2 h. The protein bands was detected using the ECL kit (Proteintech, China), and image was scanned using an imaging system (Aplegen, United States). Protein gray value was detected by ImageJ software, and then calculated the relative expression levels of proteins.

### 2.15 Statistical analyses

Data are presented as mean ± SE. All data were analyzed using the Prism 6 program (GraphPad Software, Inc.). Statistical significance was assessed using Student’s t-test. *p*-value< 0.05 was considered to be statistically significant

## 3 Results

### 3.1 Protective effect of compound K on Aβ-induced HT22 cells

**FIGURE 1:**
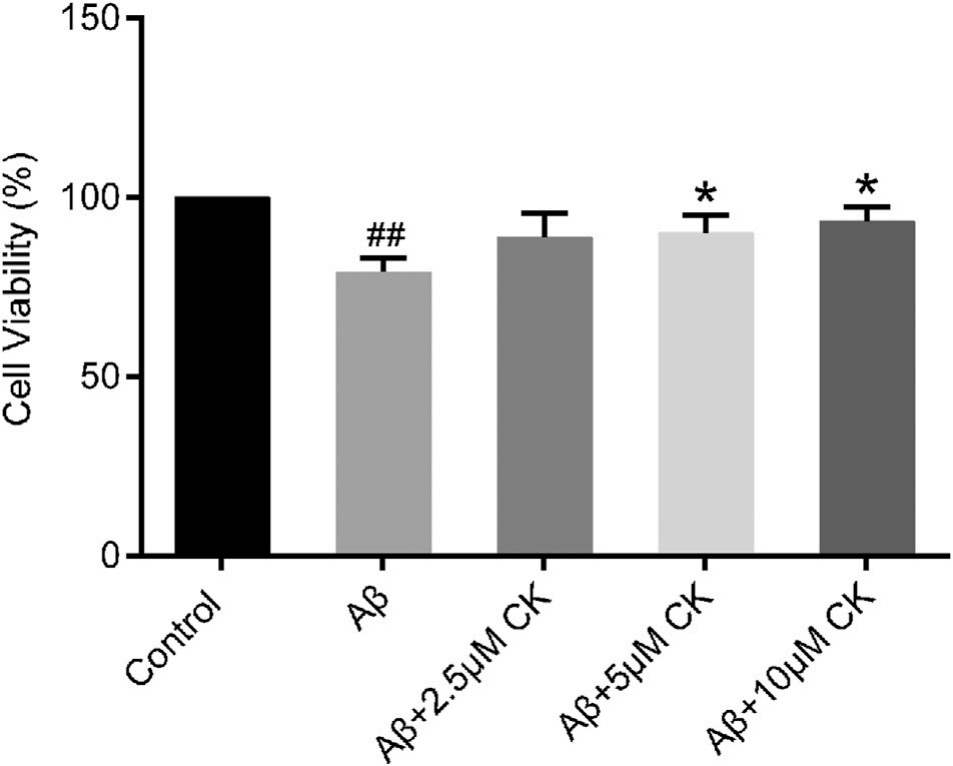
Effect of CK on Aβ-induced survival of HT22 cells. Data are presented as mean ± SE, ^#^ *p* < 0.05 and ^##^ *p* < 0.01 vs. Control, *p < 0.05 and **p < 0.01 vs. Aβ. (*n* = 8).

Aβ-treated HT22 cells showed a significant decrease in cell viability (79.15 ± 4.02) %, and increased cell viability after CK treatment. However, 2.5 μM CK was insignificant in increasing cell viability of HT22 cells, with the cell survival rate gradually increasing with an increase in the CK dose (Figure 1).

### 3.2 Regulating effect of compound K on memory ability in mice

**FIGURE 2:**
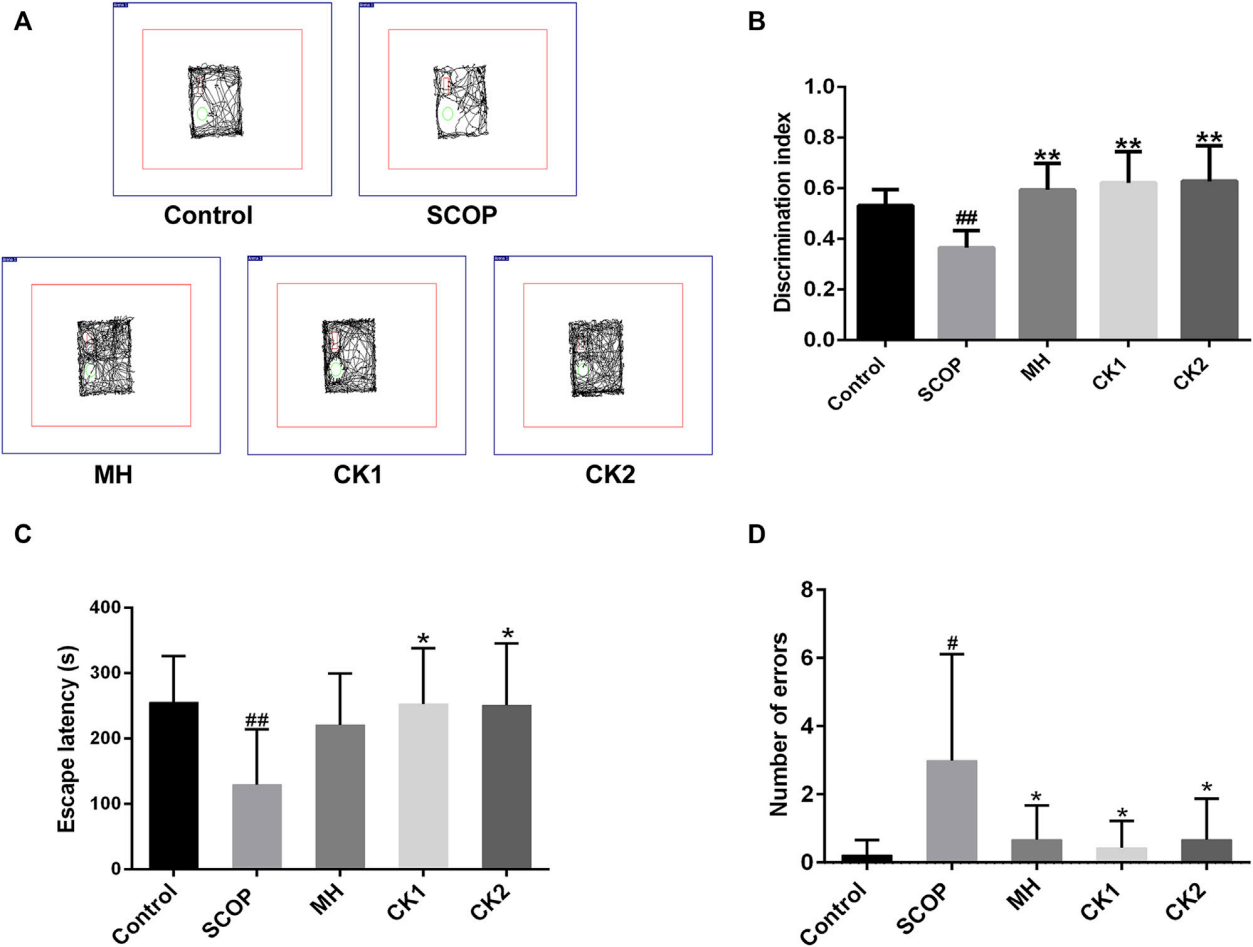
The ability of CK to regulate memory ability in mice injected with SCOP. **(A)** Representative traces of each group of mice during the testing phase. **(B)** Discrimination index of each group of mice. **(C)** Effect of CK on the latency of mice injected with SCOP. **(D)** Effect of CK on the number of errors in mice injected with SCOP. Data are presented as mean ± SE, ^#^ *p* < 0.05 and ^##^ *p* < 0.01 vs. Control, *p < 0.05 and **p < 0.01 vs. SCOP. (*n* = 8).

Mice with SCOP-induced cognitive dysfunction showed similar preference for familiar and novel objects, while mice treated with CK had significantly more exposure time to new objects than familiar objects and an increased recognition index (Figures 2A,B). The latency of mice was significantly decreased after intraperitoneal injection of SCOP, with an increased error number being observed. There was no significant increase in the latency after pre-administration of memantine hydrochloride; however, the number of errors was reduced in these mice. The latency increased after pre-administration of CK, and the number of errors also reduced (Figures 2C,D). These results indicate that CK can improve the memory of mice, with an improvement better than memantine hydrochloride being recorded.

### 3.3 Regulation of Aβ42 accumulations by compound K

**FIGURE 3:**
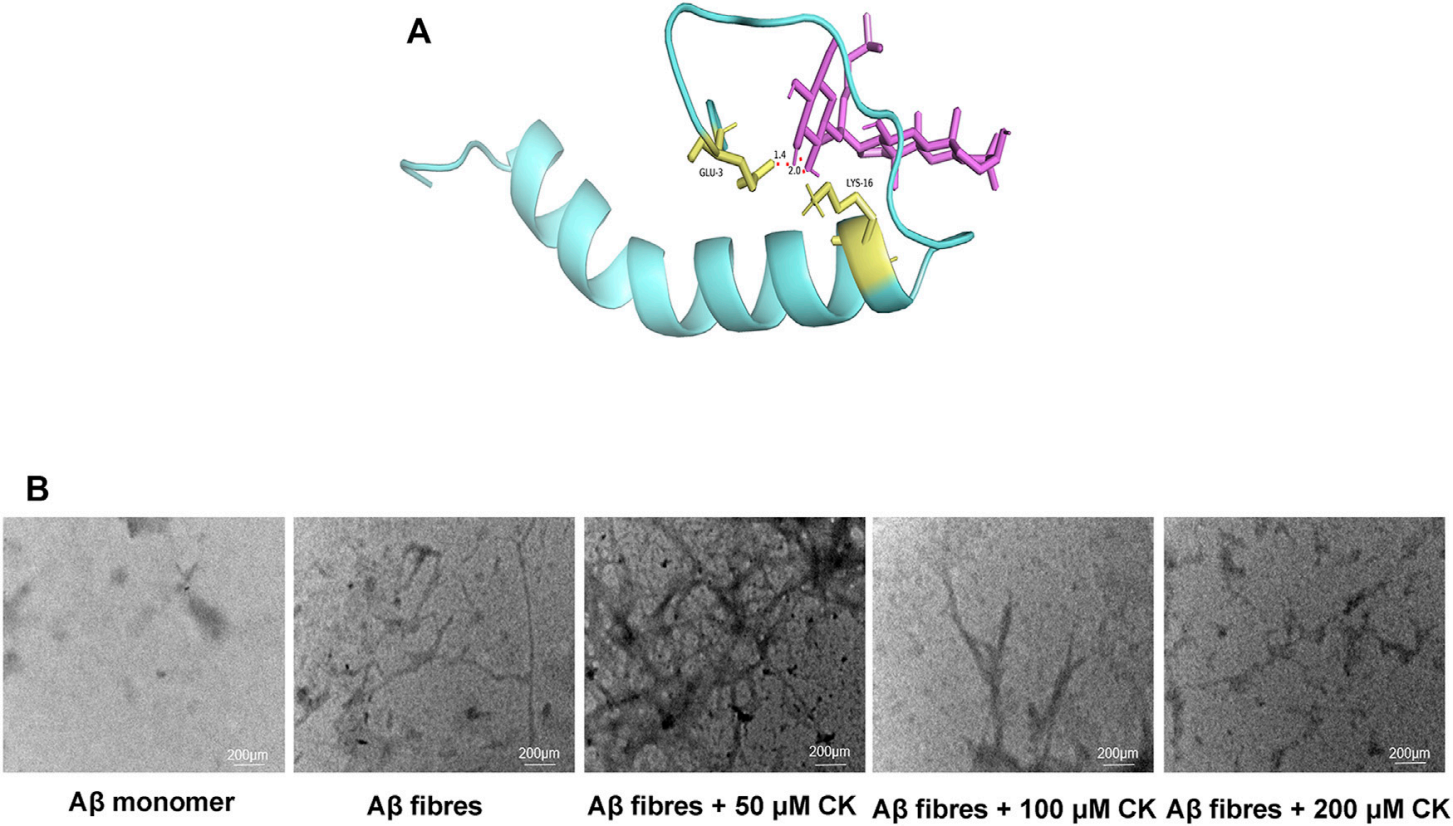
Regulation of Aβ accumulation by CK. **(A)** Molecular docking of CK and Aβ42. **(B)** The regulation of CK on the morphology of Aβ42 accumulation.

Through molecular docking, ginsenoside CK was shown to bind to Aβ by interacting with Lys16 and Glu3 of Aβ42 (Figure 3A). Unincubated Aβ monomer showed no obvious structure (Figure 3B). Aβ produced more fibrous filaments and a small amount of cluster-like structures after incubation; 50 μm CK and Aβ co-incubation did not significantly reduce the formation of filaments and cluster structures. Non-etheless, fiberous filaments and cluster structures gradually decreased after co-incubation of 100 μM or 200 μM CK with Aβ. These findings indicate that CK can inhibit the formation of Aβ aggregates.

### 3.4 Regulation of Aβ42 levels by compound K

**FIGURE 4:**
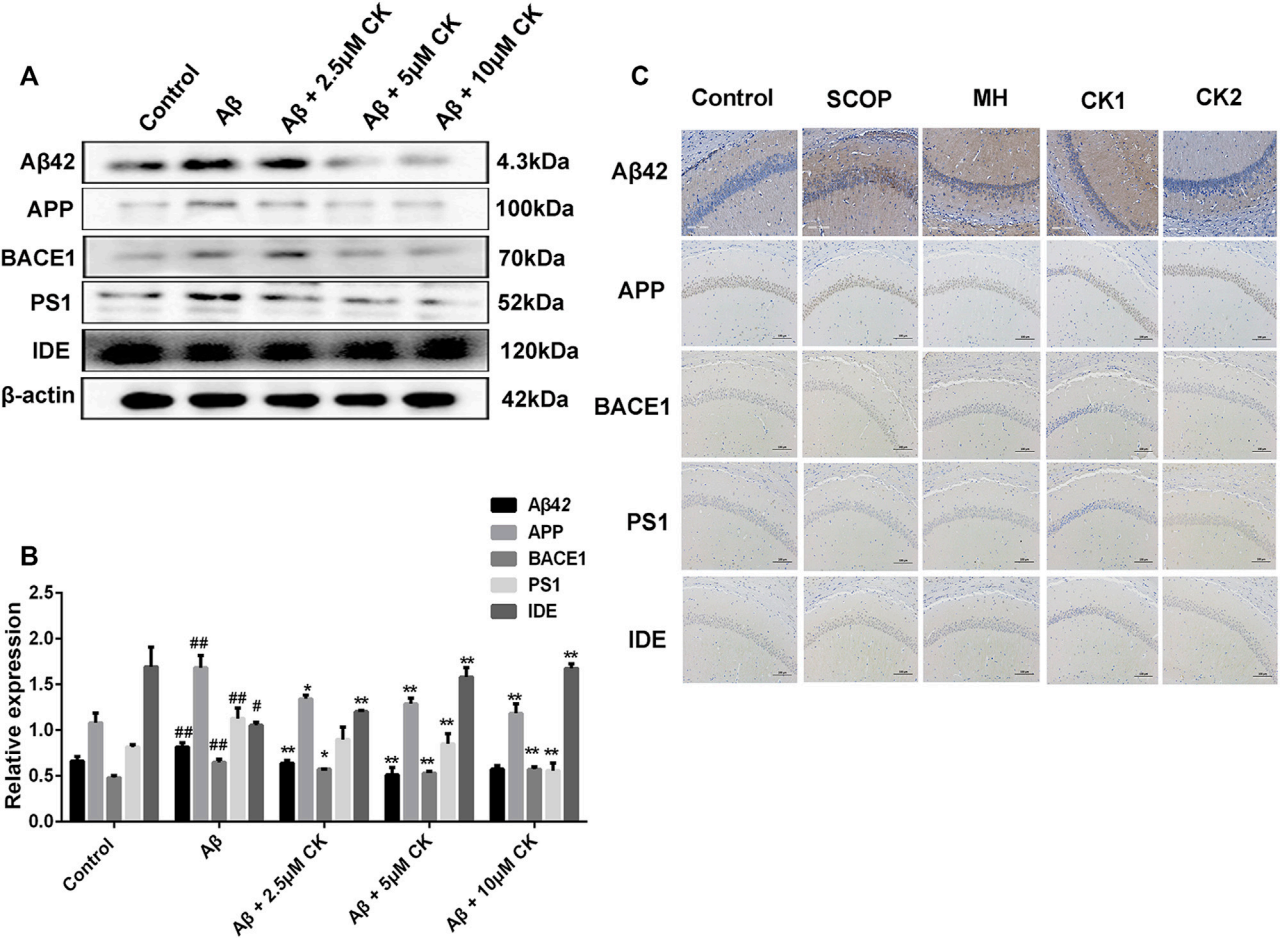
The ability of CK to regulate Aβ42 levels. **(A, B)** CK regulates the production and degradation of Aβ in neuronal HT22 cells. Data are presented as mean ± SE, ^#^ *p* < 0.05 and ^##^ *p* < 0.01 vs. Control, *p < 0.05 and **p < 0.01 vs. Aβ. **(C)** Regulatory effect of CK on Aβ expression in the hippocampus of mice. (*n* = 3).

Increased Aβ deposition plaques was observed in the hippocampus of mice after SCOP injection; however, these plaques were significantly reduced and the gray value was reduced in mice pretreated with CK. Increased expression of APP, BACE1, PS1, and Aβ, and reduced expression of IDE were observed in Aβ-injured HT22 cells and mice with SCOP-induced cognitive dysfuction. Contrarily, CK treatment decreased the expression of APP, BACE1, PS1, and Aβ, and increased that of IDE (Figure 4). These findings suggest that ginseng CK can inhibit the production of APP and further inhibit the cleavage of APP by BACE1 and PS1, produce a large number of IDE, and thus regulate the deposition of Aβ in neurons.

### 3.5 Effect of compound K on the expression of synapse-related proteins in neurons

**FIGURE 5:**
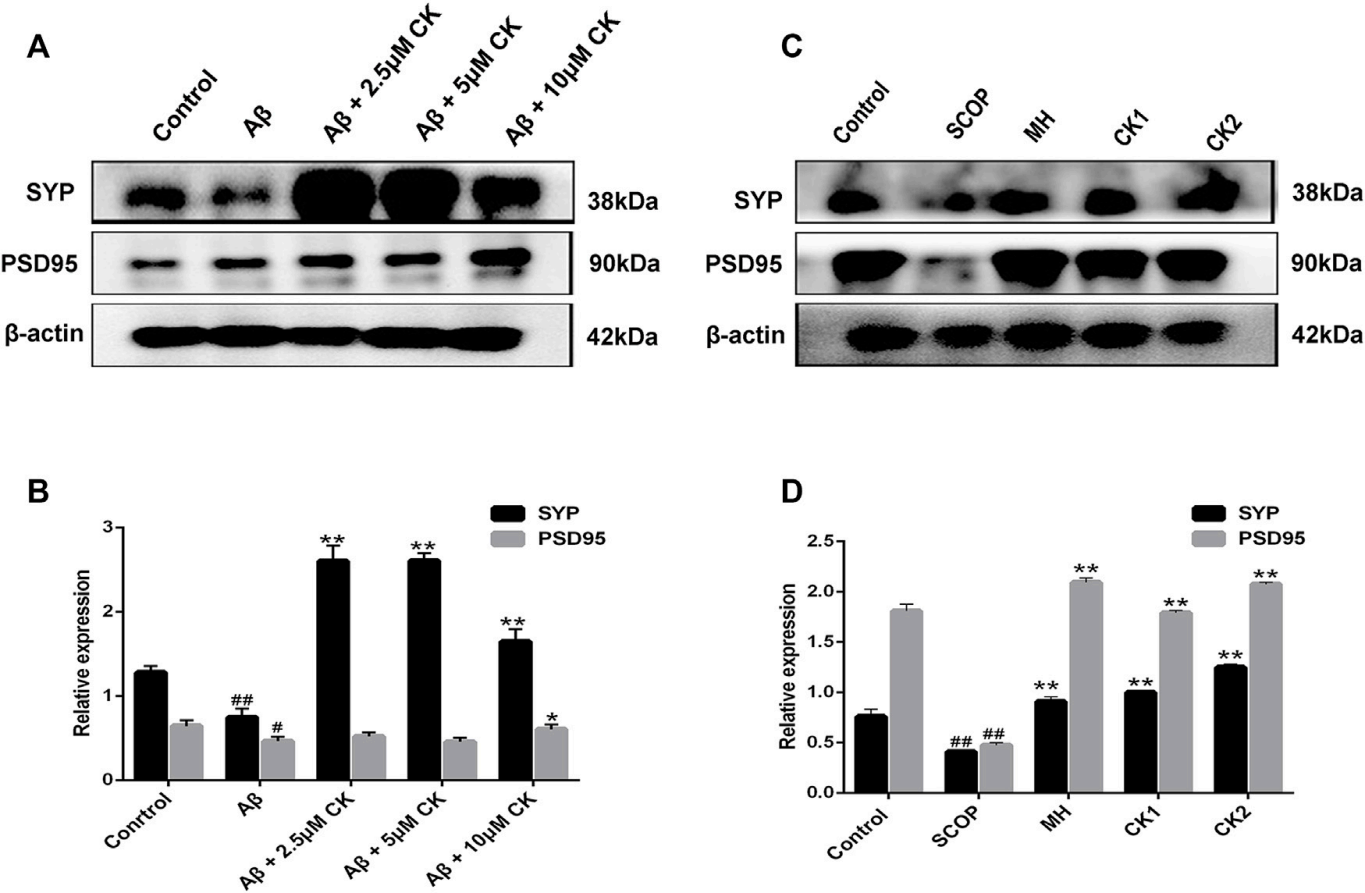
The effect of CK on synaptic plasticity in neurons. **(A, B)** Effect of CK on the regulation of SYP and PSD95 protein expression levels in Aβ-damaged HT22 cells. Data are presented as mean ± SE, ^#^ *p* < 0.05 and ^##^ *p* < 0.01 vs. Control, ^*^ *p* < 0.05 and ^**^ *p* < 0.01 vs. Aβ. **(C, D)** Effect of CK on the regulation of SYP and PSD95 protein expression levels in brain of mice injected with SCOP. Data are presented as mean ± SE, ^#^ *p* < 0.05 and ^##^ *p* < 0.01 vs. Control, *p < 0.05 and **p < 0.01 vs. SCOP. (*n* = 3).

The expression levels of SYP and PSD95 in HT22 cells after Aβ injury were significantly decreased, significantly increased SYP expression levels after pretreatment with different doses of CK, high dose of CK increases the expression level of PSD95, CK of 2.5 uM and 5 uM had no significant effect on the expression level of PSD95 in HT22 cells. The expression levels of SYP and PSD95 in the brain of mice were significantly decreased after intraperitoneal injection, pretreatment of memantine hydrochloride and CK showed a significant increase in the expression levels of SYP and PSD95 in mouse brain (Figure 5). Through *in vivo* and *in vitro* experiments, it is speculated that CK can improve synaptic function and structure of injured neurons.

### 3.6 Regulation of oxidative damage of neurons by compound K

**FIGURE 6:**
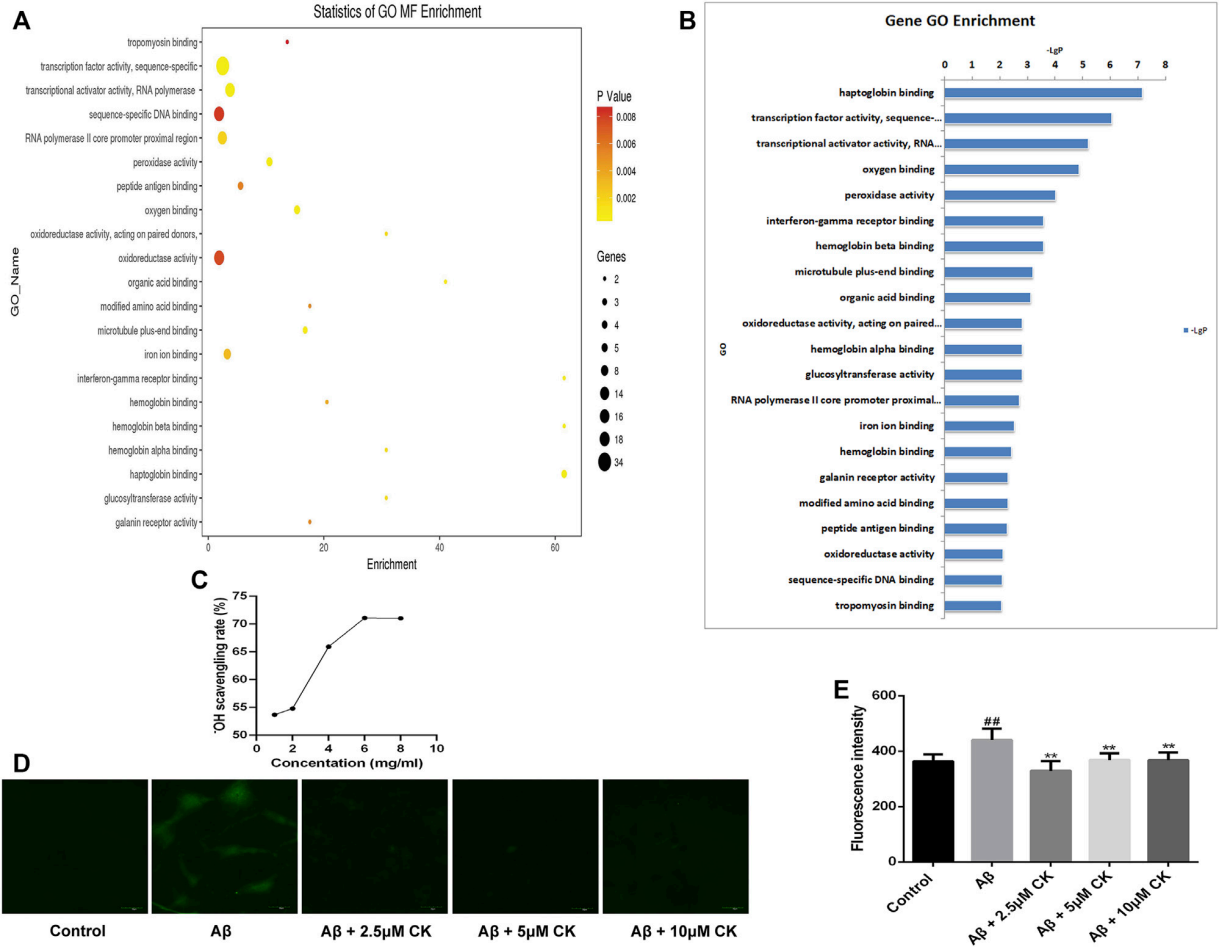
Regulation of reactive oxygen species by CK. **(A)** Statistics of GO molecular function (MF) Enrichment; the size of the circle represents the number of genes involved. The color of the circle from deep to shallow represents the *p*-value from large to small (*p* < 0.01). **(B)** Sorting molecular functions based on -LgP values (GO enrichment histogram). **(C)** Scavenging ability of CK against hydroxyl radicals. (*n* = 8) **(D, E)** Expression of ROS and fluorescence intensity in HT22 cells. (*n* = 3). Data are presented as mean ± SE, ^#^ *p* < 0.05 and ^##^ *p* < 0.01 vs. Control, *p < 0.05 and **p < 0.01 vs. Aβ.

The GO database can provide functional classification for genomic data, including categories of biological processes (BP), cellular component (CC), and molecular function (MF). Herein, we mainly performed MF analysis. The *p*-value is presented as a logarithm and has a negative value; therefore, the larger the value of (-LgP), the smaller the *p*-value, indicating a higher GO significance level. We screened out the molecular functions of 21 significant GO enrichments and, as previously reported, molecular functions such as oxygen binding, peroxidase activity, oxidoreductase activity, hemoglobin (Hb) binding, hemoglobin beta (Hb β) binding, and hemoglobin alpha (Hb α) binding had a direct or indirect relationship with the regulation of ROS (Richter et al., 2009; Al Ghouleh et al., 2011; Barman and Fulton, 2017; Altinoz et al., 2019) (Figures 6A,B). Hb and oxygen combine to form oxyhemoglobin, which increases the level of oxidative stress in neurons and increases the production of ROS (Agyemang et al., 2021). Both ROS production and scavenging are determined by oxidoreductase activity. Peroxidase, a type of oxidoreductase, is also involved in the production and clearance of ROS (Vlasova, 2018). To confirm that CK can affect the regulation of ROS, we determined its abilities to scavenge hydroxyl radicals (Figure 6C), and a fluorescent probe displayed that a large amount of ROS was produced in Aβ-injured HT22 cells. Reduction of ROS in HT22 cells was observed after pretreatment with CK (Figures 6D,E). These results further indicate that CK is involved in the regulation of ROS and reduces its production.

### 3.7 Regulation of the Nrf2/Keap1 signalling pathway by compound K

**FIGURE 7:**
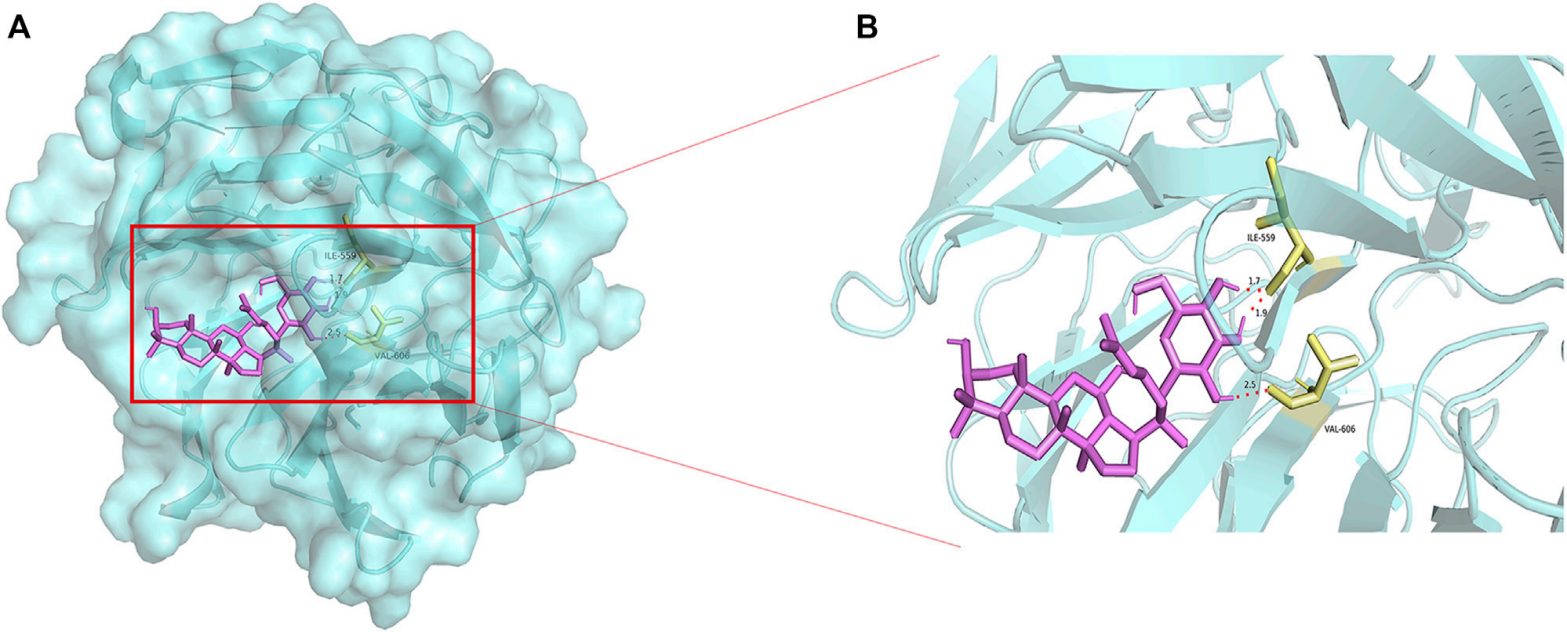
Molecular docking of CK and Nrf2-Keap1. **(A)** Docking of CK with the Nrf2-Keap1 complex. **(B)** Binding sites of CK to the Nrf2-Keap1 complex.

**FIGURE 8:**
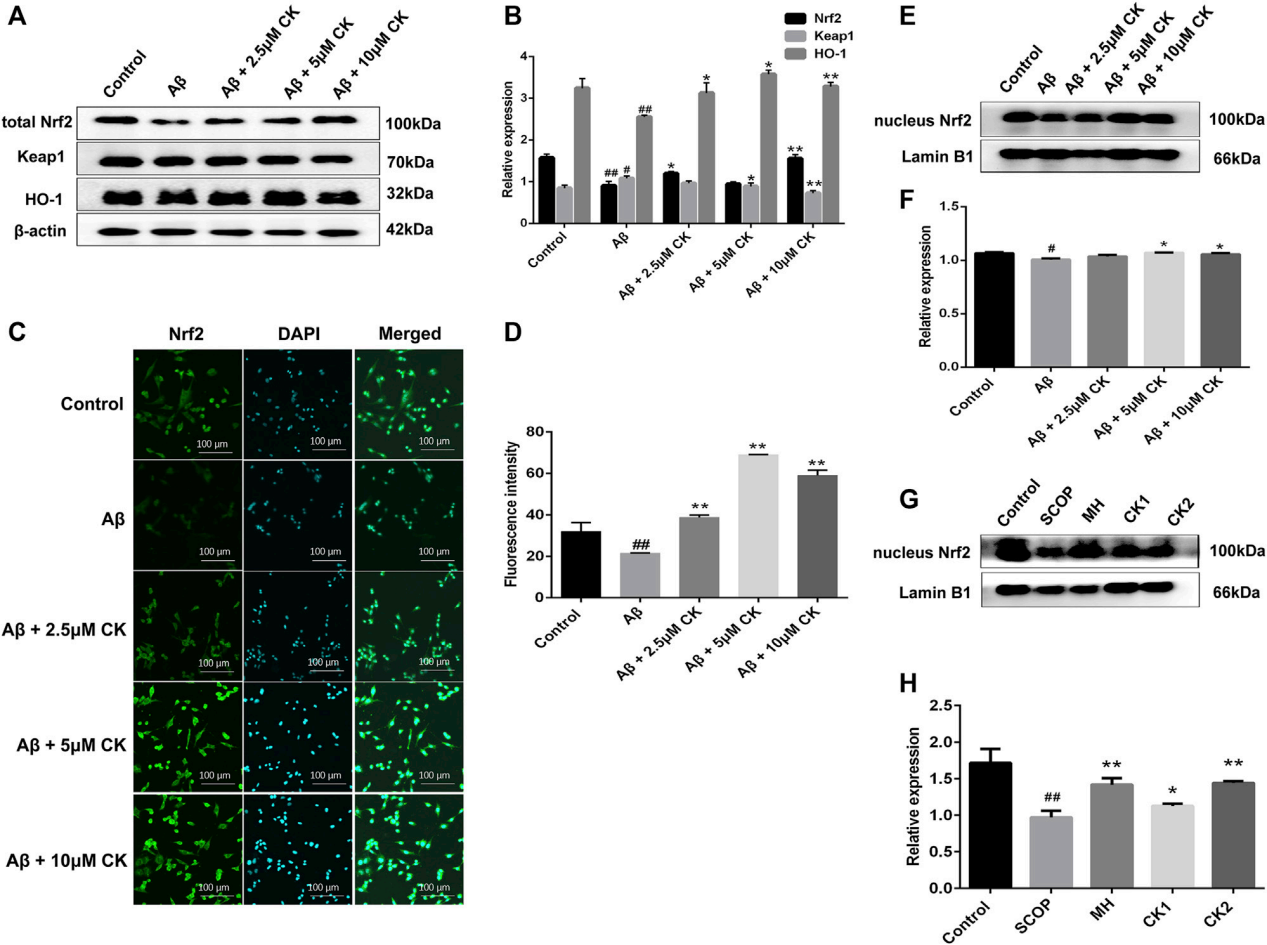
The effect of CK on the Nrf2/Keap1 signaling pathway in neurons. **(A, B)** Relative expression intensity of Nrf2/Keap1 in HT22 cells. Data are presented as mean ± SE, ^#^ *p* < 0.05 and ^##^ *p* < 0.01 vs. Control, *p < 0.05 and **p < 0.01 vs. Aβ. **(C)** Fluorescence labeled Nrf2 antibody in HT22 cells. **(D)** Fluorescence intensity of Nrf2 expression in HT22 cells. Data are presented as mean ± SE, ^#^ *p* < 0.05 and ^##^ *p* < 0.01 vs. Control, *p < 0.05 and **p < 0.01 vs. Aβ. **(E, F)** Relative expression intensity of nucleus Nrf2 in HT22 cells. Data are presented as mean ± SE, ^#^ *p* < 0.05 and ^##^ *p* < 0.01 vs. Control, *p < 0.05 and **p < 0.01 vs. Aβ. **(G, H)** Relative expression intensity of nucleus Nrf2 in mice brain. Data are presented as mean ± SE, ^#^ *p* < 0.05 and ^##^ *p* < 0.01 vs. Control, *p < 0.05 and **p < 0.01 vs. SCOP. (*n* = 3).

The overproduction of ROS generates oxidative stress, Nrf2 plays an important role in antioxidant stress (Deshmukh et al., 2017). CK molecularly bound to the Nrf2/Keap1 complex through interacting with ILE-559 and VAL 606 of Nrf2/Keap1 complex (Figure 7). Nrf2 fluorescence intensity was reduced in Aβ-injured HT22 cells, and the expression of nuclei and total Nrf2 were reduced in Aβ-injured HT22 cells and mice with SCOP-induced cognitive impairment. Non-etheless, after pretreatment with CK, the fluorescence intensity of Nrf2 in HT22 cells increased and the expression of total and nuclei Nrf2 increased. Keap1 and HO-1 act as downstream factors of Nrf2 and participate in the regulation of ROS and oxidative stress (Abed et al., 2015; Guo et al., 2015). Aβ induced the increased expression of Keap1 and decreased expression of HO-1 in HT22 cells (Figure 8). CK reversed the expression of these two factors, findings similar to a previously reported *in vivo* study (Yang et al., 2019). Our findings indicate that CK activates the Nrf2/Keap1 signaling pathway and inhibits oxidative damage in HT22 cells.

### 3.8 Inhibitory effect of compound K on neuronal apoptosis

**FIGURE 9:**
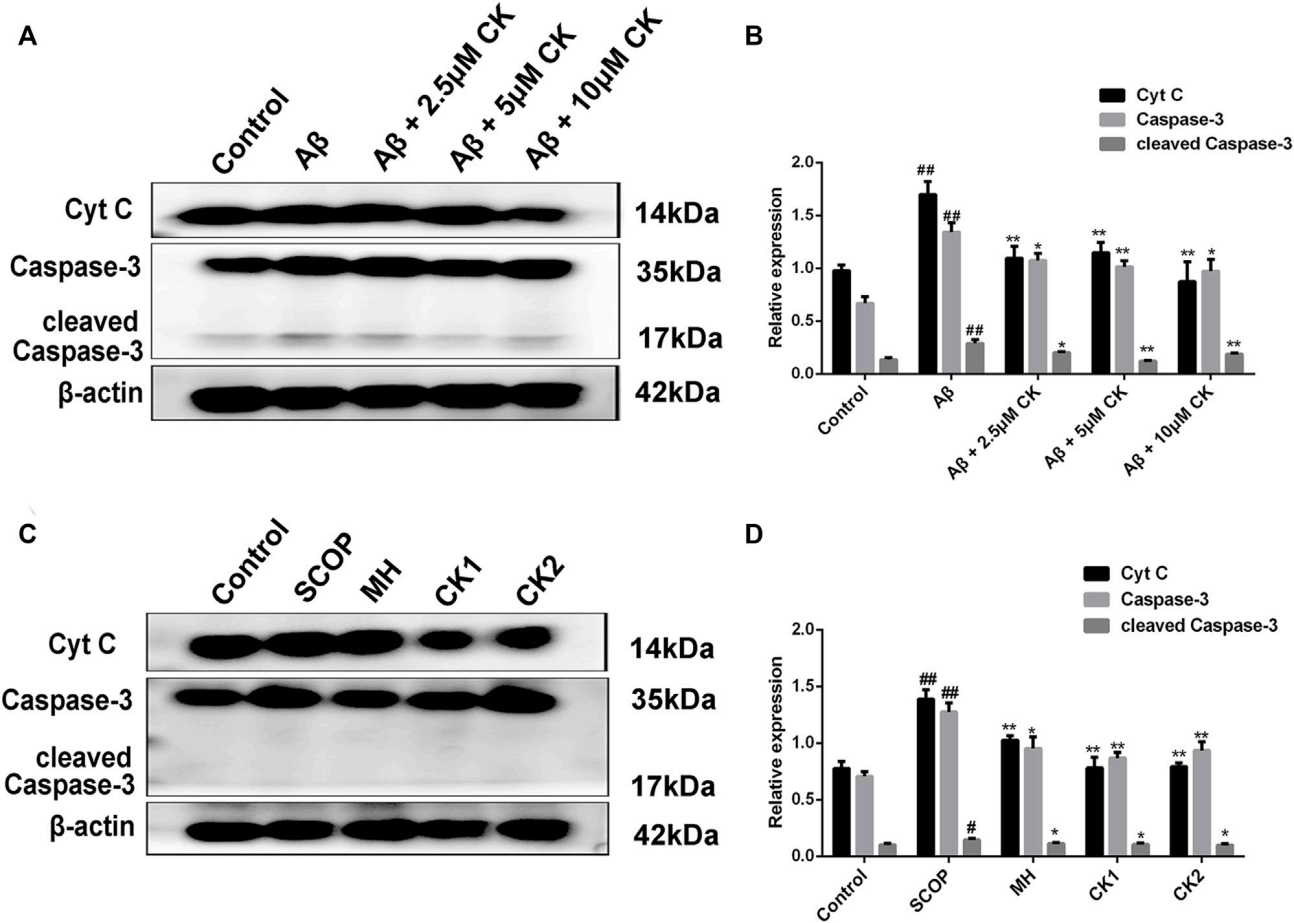
Apoptosis inhibition of CK on Aβ-damaged HT22 cells and brain of mice with SCOP-induced cognitive impairment. **(A, B)** Regulation of expression of apoptotic proteins in Aβ-injured HT22 cells by CK. Data are presented as mean ± SE, #*p* < 0.05 and ##*p* < 0.01 vs. Control, *p < 0.05 and **p < 0.01 vs. Aβ. **(C, D)** Effect of CK on apoptotic proteins in brain tissue of mice in each group. Data are presented as mean ± SE, #*p* < 0.05 and ##*p* < 0.01 vs. Control, *p < 0.05 and **p < 0.01 vs. SCOP. (*n* = 3).

An increase in the levels of proapoptotic proteins Cytochrome C (Cyt C), Caspase-3, and cleaved Caspase-3 was observed in HT22 cells after Aβ injury as well in brain tissue of mice with SCOP-induced cognitive impairment; however, these levels decreased after treatment with CK (Figure 9). To this end, CK can alleviate Aβ-damage in HT22 cells and mice with SCOP-induced cognitive impairment by inhibiting apoptosis. Further, as the dose of CK increased, the protective effect of HT22 cells was enhanced.

## 4 Discussion

Alzheimer’s disease (AD) is a progressive neurodegenerative disease. Its main pathological feature is the massive deposition of Aβ to form Aβ fibrous filaments and oligomers (Sakahira et al., 1998). In this study, a large amount of fiber structure was produced after incubation of Aβ42 monomer as observed using transmission electron microscopy. CK inhibited the formation of oligomers and fibrous structures that resulted from Aβ accumulation. Overexpression of Aβ deposition occurs *in vitro* and *in vivo* causing neurotoxicity (Wirths and Bayer, 2012), the former being investigated in HT22 cells, an immortalized mouse hippocampal neuronal cell line (Cho et al., 2015), in this study, Aβ deposition causes damage of hippocampal HT22 cells, CK can reduce the damage of Aβ to neurons. SCOP has been widely used in models of learning and memory impairment mainly including AD, and is able to induce the release of Aβ (Hernandez-Rodriguez et al., 2020; Ning et al., 2021). Treatment with SCOP may better model the increase in Aβ production that is observed during AD (Joseph et al., 2020). Therefore, in this study, the AD model was established by intraperitoneal injection of SCOP, and the treatment of CK was found to improve the learning memory ability of AD model mice.

Aβ plaques is a significant pathological factor of AD, Aβ plaques include monomers, oligomers, and fibers. Oligomers and fibers have been shown to be neurotoxic, forming through the aggregation of Aβ monomers (Söderberg et al., 2022), Aβ monomers include Aβ42 and Aβ40 monomers, Aβ42 and Aβ40 monomers are produced by anomalous cutting the amyloid precursor protein (APP) (Jiang et al., 2014). Intracellular APP forms a sAPPβ fragment and a C-terminal fragment (C99), which are cleaved by *ß*-secretase (BACE1); the C99 peptide is further secreted by γ-secretase (PS1) to secrete Aβ monomers into extracellular space (Park et al., 2018), abnormal aggregation in extracellular and intercellular space to then occurs to form Aβ oligomers and Aβ fibrous filaments (Hong et al., 2015). Aβ42 is more neurotoxic than Aβ40 (Tiwari et al., 2019), and we focused on Aβ42 monomers. In the present study, CK reduce the expression of Aβ42 monomers in neurons and inhibits expression of APP and APP cleavage enzymes (BACE1 and PS1). Under normal conditions, Aβ monomers production and clearance are in dynamic equilibrium (Baranello et al., 2015), and when the Aβ monomers clearance pathway is impeded it leads to the abnormal accumulation of Aβ monomers (Kilger et al., 2011). Therefore, improving the clearance and degradation of Aβ is a crucial link in alleviating disease progression (Song et al., 2018). Insulin degrading enzyme (IDE) is one of the important Aβ degrading enzymes (Bulloj et al., 2008). When Aβ monomers are overproduced, the cells secrete a large amount of IDE, which degrades Aβ monomers (Son et al., 2015). In this study we found that CK improved the expression of BACE1, PS1, IDE and regulated the balance of Aβ42 monomer production and clearance *in vivo* and *in vitro*. Further CK bound to Aβ42 monomer to inhibit Aβ42 aggregation and reduce Aβ fibril and oligomer production.

Abnormal deposition of Aβ causes neuronal mitochondrial disorders, thereby inducing neuronal loss and synaptic damage (Tonnies and Trushina, 2017). Synaptic impairment and synaptic loss in the brain predispose to cognitive dysfunction (Kamat et al., 2016). Post-synaptic density membrane 95 (PSD95) and synaptic vesicle protein (SYP) are important synaptic density markers (Manczak et al., 2016). PSD-95 is a scaffold protein mainly distributed in the post-synaptic density region, and is important for synaptic signal transmission and conduction (Yu et al., 2018), SYP is mainly distributed in the presynaptic membrane and is important for synaptic structure and function (Chung et al., 2019). Our study findings show that CK increases the expression levels of PSD95 and SYP in neurons, inhibits Aβ associated neuronal toxicity and synaptic dysfunction, and overall protects neurons.

Aβ plaque formation is an important pathological manifestation of AD, dysregulation of Aβ causes neurotoxicity in the early stage of AD (Raghavan et al., 2020), which is the initiating factor of AD, and later triggers oxidative stress, neuroinflammation, and apoptotic cascade responses, exacerbating neuronal damage and promoting the development of AD (Li et al., 2020). Aβ plaque induce the production of a large amount of ROS (Sharma et al., 2016), causing oxidative stress in neurons and giving rise to their oxidative damage (Reynolds et al., 2016). Our previous study found that CK can reduce oxidative damage in mouse brain tissue. GO enrichment analysis revealed that ginsenoside CK affects molecular functions such as oxygen binding, peroxidase activity, oxidoreductase activity, hemoglobin binding, hemoglobin beta binding and hemoglobin alpha binding. Hb is responsible for transporting oxygen within red blood cells (Ciaccio et al., 2022). It consists of two α- and two *ß*-subunits, each consisting of a peptide chain and a heme group (Ahmed et al., 2020). Hb α- and *ß*-globin was also found in the brain tissue of AD patients, oxygen combines with hemoglobin to form oxyhemoglobin, and autoxidation of oxyhemoglobin facilitates the release of ROS (Lu et al., 2022). Hemoglobin also binds Aβ in the neurons of patients with AD, increasing the aggregation of Aβ and accelerating ROS production (Wu et al., 2004). Eroxidase and oxidoreductase are involved in the production and scavenging of ROS. ROS include hydroxyl radical (^.^OH), hydrogen peroxide (H_2_O_2_), and superoxide anion (O_2_^.-^) (Kennedy et al., 2012). Previous studies have shown that CK inhibits the production of ROS in different tissue and cell (Huang et al., 2020; Oh and Chun, 2022). In this study, through ROS fluorescence detection and hydroxyl radical scavenging experiments, our findings further confirmed that CK inhibits the production of ROS in neurons, this is also consistent with previous studies.

Nrf2 is a transcription factor with high sensitivity to oxidative stress. It can induce the expression of multiple antioxidant proteins. In addition, it strongly inhibits the production of ROS to decrease the amount of intracellular ROS and status to maintain redox homeostasis in neurons (Kahroba et al., 2021). Under normal circumstances, Nrf2 binds to Keap1 and remains in the cytoplasm and is degraded by the proteasome (Karkkainen et al., 2014). Upon exposure to oxidative sctress, Nrf2 degradation is reduced and translocated to the nucleus, and induce the expression of HO-1 antioxidant enzymes to reduce peroxidative damage (Cui et al., 2019). We previously reported that CK promotes Nrf2 expression in mouse brain tissue (Yang et al., 2019), but the related mechanism has not been elucidated, and further studies in this study revealed that CK binds to the Nrf2-Keap1 complex, promotes the release of Nrf2, which is translocated to the nucleus of neurons, regulates the expression of downstream proteins, initiates antioxidant defense, and inhibits neuronal oxidative damage *in vitro* and *in vivo*.

Oxidative stress increases APP shear and Aβ production (Zhao and Zhao, 2013), It also promotes neuronal mitochondrial apoptosis during ROS production and massive accumulation of Aβ (Jeong et al., 2019). Cyt C, a small heme, is an important component of hemoglobin with peroxidase-like activity and is involved in the transport of oxygen (Kalpage et al., 2019; Waghwani and Douglas, 2021). Oxidized Cyt C increases ROS production, and also serves as an important mediator of apoptosis, Cyt C release from mitochondria (Ow et al., 2008), is followed by activation of Caspase-3, which further develops apoptosis (Cheng et al., 2019). In this study, CK downregulated the expression levels of Cyt C and Caspase-3 in neurons, and inhibited neuronal apoptosis induced by Aβ-induced oxidative damage.

Overall, CK may regulate the balance of Aβ monomer production and clearance by regulating APP and related shear and degradative enzymes, and binds to Aβ monomers to inhibit the formation of Aβ aggregates, which further binds to the Nrf2-Keap1 complex to promote the release of Nrf2 into the nucleus, activate the Nrf2/Keap1 signaling pathway, and reduce ROS production, thereby inhibiting Aβ aggregate-induced oxidative damage in neurons, reducing neuronal apoptosis, and improving neuronal synaptic dysfunction.

## Data Availability

The original contributions presented in the study are publicly available. The data presented in the study are deposited in the GEO repository, accession number GSE221791. https://www.ncbi.nlm.nih.gov/geo/query/acc.cgi?acc=GSE221791.

## References

[B1] Abed D. A. Goldstein M. Albanyan H. Jin H. Hu L. (2015). Discovery of direct inhibitors of Keap1-Nrf2 protein-protein interaction as potential therapeutic and preventive agents. Acta Pharm. Sin. B 5 (4), 285–299. 10.1016/j.apsb.2015.05.008 26579458 PMC4629420

[B2] Agyemang A. A. Kvist S. V. Brinkman N. Gentinetta T. Illa M. Ortenlöf N. (2021). Cell-free oxidized hemoglobin drives reactive oxygen species production and pro-inflammation in an immature primary rat mixed glial cell culture. J. Neuroinflammation 18 (1), 42. 10.1186/s12974-020-02052-4 33573677 PMC7879625

[B3] Ahmed M. H. Ghatge M. S. Safo M. K. (2020). Hemoglobin: Structure, function and allostery. Subcell. Biochem. 94, 345–382. 10.1007/978-3-030-41769-7_14 32189307 PMC7370311

[B4] Al Ghouleh I. Khoo N. K. Knaus U. G. Griendling K. K. Touyz R. M. Thannickal V. J. (2011). Oxidases and peroxidases in cardiovascular and lung disease: New concepts in reactive oxygen species signaling. Free Radic. Biol. Med. 51 (7), 1271–1288. 10.1016/j.freeradbiomed.2011.06.011 21722728 PMC3205968

[B5] Altinoz M. A. Guloksuz S. Schmidt-Kastner R. Kenis G. Ince B. Rutten B. P. F. (2019). Involvement of hemoglobins in the pathophysiology of Alzheimer's disease. Exp. Gerontol. 126, 110680. 10.1016/j.exger.2019.110680 31382012

[B6] Baranello R. J. Bharani K. L. Padmaraju V. Chopra N. Lahiri D. K. Greig N. H. (2015). Amyloid-beta protein clearance and degradation (ABCD) pathways and their role in Alzheimer's disease. Curr. Alzheimer Res. 12 (1), 32–46. 10.2174/1567205012666141218140953 25523424 PMC4820400

[B7] Barman S. A. Fulton D. (2017). Adventitial fibroblast Nox4 expression and ROS signaling in pulmonary arterial hypertension. Adv. Exp. Med. Biol. 967, 1–11. 10.1007/978-3-319-63245-2_1 29047077

[B8] Bulloj A. Leal M. C. Surace E. I. Zhang X. Xu H. Ledesma M. D. (2008). Detergent resistant membrane-associated IDE in brain tissue and cultured cells: Relevance to Abeta and insulin degradation. Mol. Neurodegener. 3, 22. 10.1186/1750-1326-3-22 19117523 PMC2648957

[B9] Cheng J. Wang H. Zhang Z. Liang K. (2019). Stilbene glycoside protects osteoblasts against oxidative damage via Nrf2/HO-1 and NF-κB signaling pathways. Arch. Med. Sci. 15 (1), 196–203. 10.5114/aoms.2018.79937 30697271 PMC6348355

[B10] Cho H. W. Jung S. Y. Lee G. H. Cho J. H. Choi I. Y. (2015). Neuroprotective effect of Citrus unshiu immature peel and nobiletin inhibiting hydrogen peroxide-induced oxidative stress in HT22 murine hippocampal neuronal cells. Pharmacogn. Mag. 11 (2), S284–S289. 10.4103/0973-1296.166047 26664016 PMC4653338

[B11] Chung J. Franklin J. F. Lee H. J. (2019). Central expression of synaptophysin and synaptoporin in nociceptive afferent subtypes in the dorsal horn. Sci. Rep. 9 (1), 4273. 10.1038/s41598-019-40967-y 30862809 PMC6414693

[B12] Ciaccio C. Coletta A. Coletta M. (2022). Role of hemoglobin structural-functional relationships in oxygen transport. Mol. Asp. Med. 84, 101022. 10.1016/j.mam.2021.101022 34509280

[B13] Cui B. Zhang S. Wang Y. Guo Y. (2019). Farrerol attenuates beta-amyloid-induced oxidative stress and inflammation through Nrf2/Keap1 pathway in a microglia cell line. Biomed. Pharmacother. 109, 112–119. 10.1016/j.biopha.2018.10.053 30396067

[B14] Deshmukh P. Unni S. Krishnappa G. Padmanabhan B. (2017). The keap1-nrf2 pathway: Promising therapeutic target to counteract ROS-mediated damage in cancers and neurodegenerative diseases. Biophys. Rev. 9 (1), 41–56. 10.1007/s12551-016-0244-4 28510041 PMC5425799

[B15] Garcia-Ptacek S. Eriksdotter M. Jelic V. Porta-Etessam J. Kareholt I. Manzano Palomo S. (2016). Subjective cognitive impairment: Towards early identification of Alzheimer disease. Neurologia 31 (8), 562–571. 10.1016/j.nrl.2013.02.007 23601758

[B16] Guo Y. Yu S. Zhang C. Kong A. N. (2015). Epigenetic regulation of Keap1-Nrf2 signaling. Free Radic. Biol. Med. 88, 337–349. 10.1016/j.freeradbiomed.2015.06.013 26117320 PMC4955581

[B17] Hernandez-Rodriguez M. Arciniega-Martinez I. M. Garcia-Marin I. D. Correa-Basurto J. Rosales-Hernandez M. C. (2020). Chronic administration of scopolamine increased GSK3βP9, beta secretase, amyloid beta, and oxidative stress in the Hippocampus of wistar rats. Mol. Neurobiol. 57 (9), 3979–3988. 10.1007/s12035-020-02009-x 32638218

[B18] Hong H. S. Maezawa I. Petrlova J. Zhao X. Y. J C. V. Jin L. W. (2015). Tomoregulin (TMEFF2) binds alzheimer's disease amyloid-β (Aβ) oligomer and AβPP and protects neurons from aβ-induced toxicity. J. Alzheimers Dis. 48 (3), 731–743. 10.3233/jad-150318 26402097 PMC5533101

[B19] Huang Q. Lou T. Wang M. Xue L. Lu J. Zhang H. (2020). Compound K inhibits autophagy-mediated apoptosis induced by oxygen and glucose deprivation/reperfusion via regulating AMPK-mTOR pathway in neurons. Life Sci. 254, 117793. 10.1016/j.lfs.2020.117793 32416164

[B20] Inestrosa N. C. Tapia-Rojas C. Griffith T. N. Carvajal F. J. Benito M. J. Rivera-Dictter A. (2011). Tetrahydrohyperforin prevents cognitive deficit, Aβ deposition, tau phosphorylation and synaptotoxicity in the APPswe/psen1de9 model of alzheimer's disease: A possible effect on APP processing. Transl. Psychiatry 1, e20. 10.1038/tp.2011.19 22832522 PMC3309512

[B21] Jeong J. Y. Cha H. J. Choi E. O. Kim C. H. Kim G. Y. Yoo Y. H. (2019). Activation of the Nrf2/HO-1 signaling pathway contributes to the protective effects of baicalein against oxidative stress-induced DNA damage and apoptosis in HEI193 Schwann cells. Int. J. Med. Sci. 16 (1), 145–155. 10.7150/ijms.27005 30662338 PMC6332480

[B22] Jiang S. Li Y. Zhang X. Bu G. Xu H. Zhang Y. W. (2014). Trafficking regulation of proteins in Alzheimer's disease. Mol. Neurodegener. 9, 6. 10.1186/1750-1326-9-6 24410826 PMC3891995

[B23] Joseph E. Villalobos-Acosta D. Torres-Ramos M. A. Farfán-García E. D. Gómez-López M. Miliar-García Á. (2020). Neuroprotective effects of apocynin and galantamine during the chronic administration of scopolamine in an alzheimer's disease model. J. Mol. Neurosci. 70 (2), 180–193. 10.1007/s12031-019-01426-5 31768942

[B24] Kahroba H. Ramezani B. Maadi H. Sadeghi M. R. Jaberie H. Ramezani F. (2021). The role of Nrf2 in neural stem/progenitors cells: From maintaining stemness and self-renewal to promoting differentiation capability and facilitating therapeutic application in neurodegenerative disease. Ageing Res. Rev. 65, 101211. 10.1016/j.arr.2020.101211 33186670

[B25] Kalpage H. A. Bazylianska V. Recanati M. A. Fite A. Liu J. Wan J. (2019). Tissue-specific regulation of cytochrome c by post-translational modifications: Respiration, the mitochondrial membrane potential, ROS, and apoptosis. FASEB J. 33 (2), 1540–1553. 10.1096/fj.201801417R 30222078 PMC6338631

[B26] Kamat P. K. Kalani A. Rai S. Swarnkar S. Tota S. Nath C. (2016). Mechanism of oxidative stress and synapse dysfunction in the pathogenesis of alzheimer's disease: Understanding the therapeutics strategies. Mol. Neurobiol. 53 (1), 648–661. 10.1007/s12035-014-9053-6 25511446 PMC4470891

[B27] Karkkainen V. Pomeshchik Y. Savchenko E. Dhungana H. Kurronen A. Lehtonen S. (2014). Nrf2 regulates neurogenesis and protects neural progenitor cells against Aβ toxicity. Stem Cells 32 (7), 1904–1916. 10.1002/stem.1666 24753106

[B28] Kennedy K. A. Sandiford S. D. Skerjanc I. S. Li S. S. (2012). Reactive oxygen species and the neuronal fate. Cell Mol. Life Sci. 69 (2), 215–221. 10.1007/s00018-011-0807-2 21947442 PMC11114775

[B29] Kilger E. Buehler A. Woelfing H. Kumar S. Kaeser S. A. Nagarathinam A. (2011). BRI2 protein regulates beta-amyloid degradation by increasing levels of secreted insulin-degrading enzyme (IDE). J. Biol. Chem. 286 (43), 37446–37457. 10.1074/jbc.M111.288373 21873424 PMC3199491

[B30] Kim E. H. Kim W. (2018). An insight into ginsenoside metabolite compound K as a potential tool for skin disorder. Evid. Based Complement. Altern. Med. 2018, 8075870. 10.1155/2018/8075870 PMC6036801 30046346

[B31] Lee B. H. Hwang S. H. Choi S. H. Kim H. J. Lee J. H. Lee S. M. (2013). Inhibitory effects of ginsenoside metabolites, compound K and protopanaxatriol, on GABAC receptor-mediated ion currents. Korean J. Physiol. Pharmacol. 17 (2), 127–132. 10.4196/kjpp.2013.17.2.127 23626474 PMC3634089

[B32] Li Y. Zhang J. Wan J. Liu A. Sun J. (2020). Melatonin regulates Aβ production/clearance balance and Aβ neurotoxicity: A potential therapeutic molecule for alzheimer's disease. Biomed. Pharmacother. 132, 110887. 10.1016/j.biopha.2020.110887 33254429

[B33] Lichtenthaler S. F. (2012). Alpha-secretase cleavage of the amyloid precursor protein: Proteolysis regulated by signaling pathways and protein trafficking. Curr. Alzheimer Res. 9 (2), 165–177. 10.2174/156720512799361655 21605033

[B34] Lu C. Dong L. Lv J. Wang Y. Fan B. Wang F. (2018). 20(S)-protopanaxadiol (PPD) alleviates scopolamine-induced memory impairment via regulation of cholinergic and antioxidant systems, and expression of Egr-1, c-Fos and c-Jun in mice. Chem. Biol. Interact. 279, 64–72. 10.1016/j.cbi.2017.11.008 29133030

[B35] Lu Y. Wang J. Tang F. Pratap U. P. Sareddy G. R. Dhandapani K. M. (2022). Regulation and role of neuron-derived hemoglobin in the mouse Hippocampus. Int. J. Mol. Sci. 23 (10), 5360. 10.3390/ijms23105360 35628182 PMC9140924

[B36] Mancuso C. Santangelo R. (2017). Panax ginseng and Panax quinquefolius: From pharmacology to toxicology. Food Chem. Toxicol. 107, 362–372. 10.1016/j.fct.2017.07.019 28698154 PMC7116968

[B37] Manczak M. Kandimalla R. Fry D. Sesaki H. Reddy P. H. (2016). Protective effects of reduced dynamin-related protein 1 against amyloid beta-induced mitochondrial dysfunction and synaptic damage in Alzheimer's disease. Hum. Mol. Genet. 25 (23), 5148–5166. 10.1093/hmg/ddw330 27677309 PMC6078633

[B38] Ning F. Chen L. Chen L. Liu X. Zhu Y. Hu J. (2021). Combination of polygoni multiflori radix praeparata and acori tatarinowii rhizoma alleviates learning and memory impairment in scopolamine-treated mice by regulating synaptic-related proteins. Front. Pharmacol. 12, 679573. 10.3389/fphar.2021.679573 34393775 PMC8360279

[B39] Oh J. Kim J. S. (2016). Compound K derived from ginseng: Neuroprotection and cognitive improvement. Food Funct. 7 (11), 4506–4515. 10.1039/c6fo01077f 27801453

[B40] Oh J. M. Chun S. (2022). Ginsenoside CK inhibits the early stage of adipogenesis via the AMPK, MAPK, and AKT signaling pathways. Antioxidants (Basel) 11 (10), 1890. 10.3390/antiox11101890 36290613 PMC9598147

[B41] Ow Y. P. Green D. R. Hao Z. Mak T. W. (2008). Cytochrome c: Functions beyond respiration. Nat. Rev. Mol. Cell Biol. 9 (7), 532–542. 10.1038/nrm2434 18568041

[B42] Park J. Kwon J. H. Kim N. Song K. (2018). Effects of 1950 MHz radiofrequency electromagnetic fields on Aβ processing in human neuroblastoma and mouse hippocampal neuronal cells. J. Radiat. Res. 59 (1), 18–26. 10.1093/jrr/rrx045 29040655 PMC5778507

[B43] Raghavan N. S. Dumitrescu L. Mormino E. Mahoney E. R. Lee A. J. Gao Y. (2020). Association between common variants in RBFOX1, an RNA-binding protein, and brain amyloidosis in early and preclinical alzheimer disease. JAMA Neurol. 77 (10), 1288–1298. 10.1001/jamaneurol.2020.1760 32568366 PMC7309575

[B44] Reynolds M. R. Singh I. Azad T. D. Holmes B. B. Verghese P. B. Dietrich H. H. (2016). Heparan sulfate proteoglycans mediate Aβ-induced oxidative stress and hypercontractility in cultured vascular smooth muscle cells. Mol. Neurodegener. 11, 9. 10.1186/s13024-016-0073-8 26801396 PMC4722750

[B45] Richter F. Meurers B. H. Zhu C. Medvedeva V. P. Chesselet M. F. (2009). Neurons express hemoglobin alpha- and beta-chains in rat and human brains. J. Comp. Neurol. 515 (5), 538–547. 10.1002/cne.22062 19479992 PMC3123135

[B46] Sadigh-Eteghad S. Sabermarouf B. Majdi A. Talebi M. Farhoudi M. Mahmoudi J. (2015). Amyloid-beta: A crucial factor in alzheimer's disease. Med. Princ. Pract. 24 (1), 1–10. 10.1159/000369101 PMC5588216 25471398

[B47] Sakahira H. Enari M. Nagata S. (1998). Cleavage of CAD inhibitor in CAD activation and DNA degradation during apoptosis. Nature 391 (6662), 96–99. 10.1038/34214 9422513

[B48] Sharma S. Verma S. Kapoor M. Saini A. Nehru B. (2016). Alzheimer's disease like pathology induced six weeks after aggregated amyloid-beta injection in rats: Increased oxidative stress and impaired long-term memory with anxiety-like behavior. Neurol. Res. 38 (9), 838–850. 10.1080/01616412.2016.1209337 27431920

[B49] Smith I. Williamson E. M. Putnam S. Farrimond J. Whalley B. J. (2014). Effects and mechanisms of ginseng and ginsenosides on cognition. Nutr. Rev. 72 (5), 319–333. 10.1111/nure.12099 24666107

[B50] Söderberg L. Johannesson M. Nygren P. Laudon H. Eriksson F. Osswald G. (2022). Lecanemab, aducanumab, and gantenerumab - binding profiles to different forms of amyloid-beta might explain efficacy and side effects in clinical trials for alzheimer's disease. Neurotherapeutics. 10.1007/s13311-022-01308-6 PMC10119362 36253511

[B51] Son S. M. Kang S. Choi H. Mook-Jung I. (2015). Statins induce insulin-degrading enzyme secretion from astrocytes via an autophagy-based unconventional secretory pathway. Mol. Neurodegener. 10, 56. 10.1186/s13024-015-0054-3 26520569 PMC4628355

[B52] Song E. S. Rodgers D. W. Hersh L. B. (2018). Insulin-degrading enzyme is not secreted from cultured cells. Sci. Rep. 8 (1), 2335. 10.1038/s41598-018-20597-6 29402917 PMC5799172

[B53] Spoelgen R. von Arnim C. A. Thomas A. V. Peltan I. D. Koker M. Deng A. (2006). Interaction of the cytosolic domains of sorLA/LR11 with the amyloid precursor protein (APP) and beta-secretase beta-site APP-cleaving enzyme. J. Neurosci. 26 (2), 418–428. 10.1523/jneurosci.3882-05.2006 16407538 PMC6674411

[B54] Takahashi K. Niidome T. Akaike A. Kihara T. Sugimoto H. (2008). Phosphorylation of amyloid precursor protein (APP) at Tyr687 regulates APP processing by alpha- and gamma-secretase. Biochem. Biophys. Res. Commun. 377 (2), 544–549. 10.1016/j.bbrc.2008.10.013 18854169

[B55] Tiwari S. Atluri V. Kaushik A. Yndart A. Nair M. (2019). Alzheimer's disease: Pathogenesis, diagnostics, and therapeutics. Int. J. Nanomedicine 14, 5541–5554. 10.2147/ijn.S200490 31410002 PMC6650620

[B56] Tonnies E. Trushina E. (2017). Oxidative stress, synaptic dysfunction, and alzheimer's disease. J. Alzheimers Dis. 57 (4), 1105–1121. 10.3233/JAD-161088 28059794 PMC5409043

[B57] Tyan S. H. Shih A. Y. Walsh J. J. Maruyama H. Sarsoza F. Ku L. (2012). Amyloid precursor protein (APP) regulates synaptic structure and function. Mol. Cell Neurosci. 51 (1-2), 43–52. 10.1016/j.mcn.2012.07.009 22884903 PMC3538857

[B58] Vlasova II (2018). Peroxidase activity of human hemoproteins: Keeping the fire under control. Molecules 23 (10), 2561. 10.3390/molecules23102561 30297621 PMC6222727

[B59] Waghwani H. K. Douglas T. (2021). Cytochrome C with peroxidase-like activity encapsulated inside the small DPS protein nanocage. J. Mater Chem. B 9 (14), 3168–3179. 10.1039/d1tb00234a 33885621

[B60] West S. Bhugra P. (2015). Emerging drug targets for Aβ and tau in alzheimer's disease: A systematic review. Br. J. Clin. Pharmacol. 80 (2), 221–234. 10.1111/bcp.12621 25753046 PMC4541970

[B61] Wirths O. Bayer T. A. (2012). Intraneuronal Aβ accumulation and neurodegeneration: Lessons from transgenic models. Life Sci. 91 (23-24), 1148–1152. 10.1016/j.lfs.2012.02.001 22401905

[B62] Wu C. W. Liao P. C. Yu L. Wang S. T. Chen S. T. Wu C. M. (2004). Hemoglobin promotes Abeta oligomer formation and localizes in neurons and amyloid deposits. Neurobiol. Dis. 17 (3), 367–377. 10.1016/j.nbd.2004.08.014 15571973

[B63] Yang Q. Lin J. Zhang H. Liu Y. Kan M. Xiu Z. (2019). Ginsenoside compound K regulates amyloid beta via the nrf2/keap1 signaling pathway in mice with scopolamine hydrobromide-induced memory impairments. J. Mol. Neurosci. 67 (1), 62–71. 10.1007/s12031-018-1210-3 30535776

[B64] Yang X. D. Yang Y. Y. Ouyang D. S. Yang G. P. (2015). A review of biotransformation and pharmacology of ginsenoside compound K. Fitoterapia 100, 208–220. 10.1016/j.fitote.2014.11.019 25449425

[B65] Yu Y. Jans D. C. Winblad B. Tjernberg L. O. Schedin-Weiss S. (2018). Neuronal Aβ42 is enriched in small vesicles at the presynaptic side of synapses. Life Sci. Alliance 1 (3), e201800028. 10.26508/lsa.201800028 30456353 PMC6238618

[B66] Zhao Y. Zhao B. (2013). Oxidative stress and the pathogenesis of Alzheimer's disease. Oxid. Med. Cell Longev. 2013, 316523. 10.1155/2013/316523 23983897 PMC3745981

